# Case Report: High-altitude exposure and severe respiratory infection precipitating diabetic ketoacidosis: new-onset ketosis-prone diabetes unmasked by physiological stress

**DOI:** 10.3389/fmed.2026.1762878

**Published:** 2026-02-26

**Authors:** Killen H. Briones-Claudett, Jaime Benites-Solis, Patricia Delgado-Cedeño, Fabián Alfonso Ortiz-Herbener, Anahí D. Briones-Zamora, Diana C. Briones-Márquez, Michelle Grunauer, Killen H. Briones-Zamora

**Affiliations:** 1 Facultad de Ciencias Médicas, de la Salud y de la Vida, Universidad Internacional del Ecuador (UIDE), Quito, Ecuador; 2 Briones PulmoCare, Guayaquil, Ecuador; 3 Omni Hospital, Guayaquil, Ecuador; 4 Instituto Ecuatoriano del Corazón (IECOREC), Guayaquil, Ecuador; 5 Unidad de Cuidado Renal Avanzado (UCRA), Guayaquil, Ecuador; 6 Instituto Universitario Italiano de Rosario (IUNIR), Rosario, Argentina; 7 Universidad San Francisco de Quito (USFQ), Quito, Ecuador; 8 Universidad de Especialidades Espíritu Santo, Facultad de Ciencias Médicas, Samborondón, Ecuador

**Keywords:** antimicrobial stewardship, bronchoscopy, diabetic ketoacidosis, high altitude, ketosis-prone diabetes, molecular diagnostics, polymicrobial pneumonia, travel medicine

## Abstract

**Background:**

Diabetic ketoacidosis (DKA) may represent the first presentation of previously unrecognized diabetes, with acute environmental and infectious stressors lowering the threshold for ketoacidosis. High-altitude hypoxia can impair glucose homeostasis and host defenses, potentially predisposing to severe respiratory infections.

**Case presentation:**

A previously undiagnosed 28-year-old woman from sea level developed DKA 1 week after returning from a 5-day trip to Cusco, Peru (3,400 m). She presented with altered mental status, Kussmaul breathing, and severe metabolic acidosis (pH 7.09, glucose 548 mg/dL, bicarbonate 6.1 mmol/L, anion gap 26 mEq/L) in the setting of progressive respiratory symptoms and hypoxemia. Chest CT showed multifocal consolidations with cylindrical bronchiectasis and air trapping. Bronchoscopy with bronchoalveolar lavage revealed thick purulent secretions, and multiplex PCR identified parainfluenza virus, rhinovirus/enterovirus, *Haemophilus influenzae*, *Staphylococcus aureus*, *Moraxella catarrhalis*, and *Candida albicans*. Highly sensitive panels can detect colonization or shedding; therefore, results require syndrome-level correlation and stewardship-based interpretation. She required mechanical ventilation for 4 days. Standard DKA management and targeted antimicrobial therapy led to resolution of ketoacidosis within 48 h. Admission HbA1c was 9.0%, supporting antecedent chronic dysglycemia rather than isolated stress hyperglycemia. The combination of negative diabetes autoantibodies, preserved C-peptide (2.1 ng/mL), and subsequent insulin independence was most consistent with an A^−^β^+^ ketosis-prone diabetes phenotype unmasked by acute stress. Pulmonary function and diffusing capacity fully recovered by 6 months.

**Conclusion:**

This case highlights DKA precipitated by recent high-altitude exposure and severe polymicrobial pneumonia in the setting of previously unrecognized chronic dysglycemia, consistent with ketosis-prone diabetes. It underscores the diagnostic value of early bronchoscopy with molecular pathogen detection and the need for stewardship-based interpretation of multiplex PCR results in complex metabolic–respiratory presentations.

## Introduction

Diabetic ketoacidosis (DKA) is a life-threatening hyperglycemic emergency caused by absolute or relative insulin deficiency, characterized by metabolic acidosis and ketonemia, and most commonly triggered by acute physiologic stress such as infection, dehydration, or trauma (1). DKA may be the first clinical presentation of diabetes in adults, including ketosis-prone diabetes (KPD), in which patients present with DKA but may later achieve glycemic remission as β-cell function recovers (2).

High-altitude hypoxia is associated with complex and heterogeneous changes in glucose homeostasis (3–5). Acute hypobaric hypoxia can increase sympathetic activation and counter-regulatory hormones, worsening insulin resistance and promoting lipolysis and ketogenesis; in susceptible individuals, altitude exposure may therefore unmask previously unrecognized dysglycemia (5, 6). Hypoxia-inducible signaling has also been implicated in impaired pancreatic β-cell function, providing a cellular mechanism that may lower the threshold for ketoacidosis during hypoxic stress (7).

Moreover, high-altitude environments can alter immune cell populations and host defenses, potentially increasing vulnerability to respiratory infections (8). Concomitant viral and bacterial respiratory infections are well-known precipitants of metabolic decompensation and can drive severe systemic inflammation that accelerates ketoacidosis (9, 10). Accordingly, the convergence of hypoxic exposure, dehydration, and severe infection may serve as a catalyst for DKA in individuals with underlying but previously unrecognized chronic dysglycemia (11).

This case presents a unique confluence of factors—recent high-altitude travel, polymicrobial respiratory infection, and sudden onset of DKA—as the first manifestation of a ketosis-prone diabetes phenotype suggested by an elevated admission HbA1c and subsequent remission with preserved endogenous insulin secretion (2, 11). Molecular testing via multiplex PCR supported early etiologic clarification and antimicrobial targeting, allowing for timely resolution of both metabolic and respiratory derangements (12, 13).

## Case presentation

A previously healthy 28-year-old woman from Guayaquil, Ecuador (approximately 0 m altitude) presented to the emergency department with altered mental status, tachypnea, and dehydration, 7 days after returning from a five-day trip to Cusco, Peru (3,400 m). While at altitude, she experienced mild cold-like symptoms but remained physically active. Anthropometric measurements at admission revealed a BMI of 24.8 kg/m^2^ and a waist circumference of 78 cm. There was no family history of early-onset diabetes, recurrent ketosis, or other endocrinopathies across multiple generations; although MODY was considered given her age and presentation, the absence of familial clustering, lack of syndromic features, and subsequent glycemic normalization with insulin independence indicated low pre-test probability, and genetic testing was not pursued (14).

After returning to sea level, she developed cough, sore throat, and fever, which progressed over 4 days to polydipsia, polyuria, fatigue, and dyspnea. On presentation, vital signs were: heart rate 122 bpm, respiratory rate 30/min, blood pressure 145/90 mmHg, and SpO₂ 89% on room air. Glasgow Coma Scale was 13/15. Physical examination revealed dry mucosa, reduced skin turgor, and bilateral basal crackles. A systematic evaluation for immune dysfunction was performed; she denied any history of recurrent, severe, unusual, or opportunistic infections and had no known immunodeficiency, no recent corticosteroid exposure, and no immunomodulatory therapy.

Laboratory testing confirmed diabetic ketoacidosis: pH 7.09, glucose 548 mg/dL, bicarbonate 6.1 mmol/L, anion gap 26 mEq/L, and ketonuria. Inflammatory markers were elevated (WBC 17,890/μL, C-reactive protein 25.3 mg/L). HbA1c was 9.0%. Autoimmune antibodies (GAD65, IA-2, ZnT8) were negative, and C-peptide level was preserved at 2.1 ng/mL, consistent with retained endogenous insulin secretion in the setting of acute metabolic decompensation, supporting a ketosis-prone diabetes phenotype rather than autoimmune type 1 diabetes (2).

**Figure 1:**
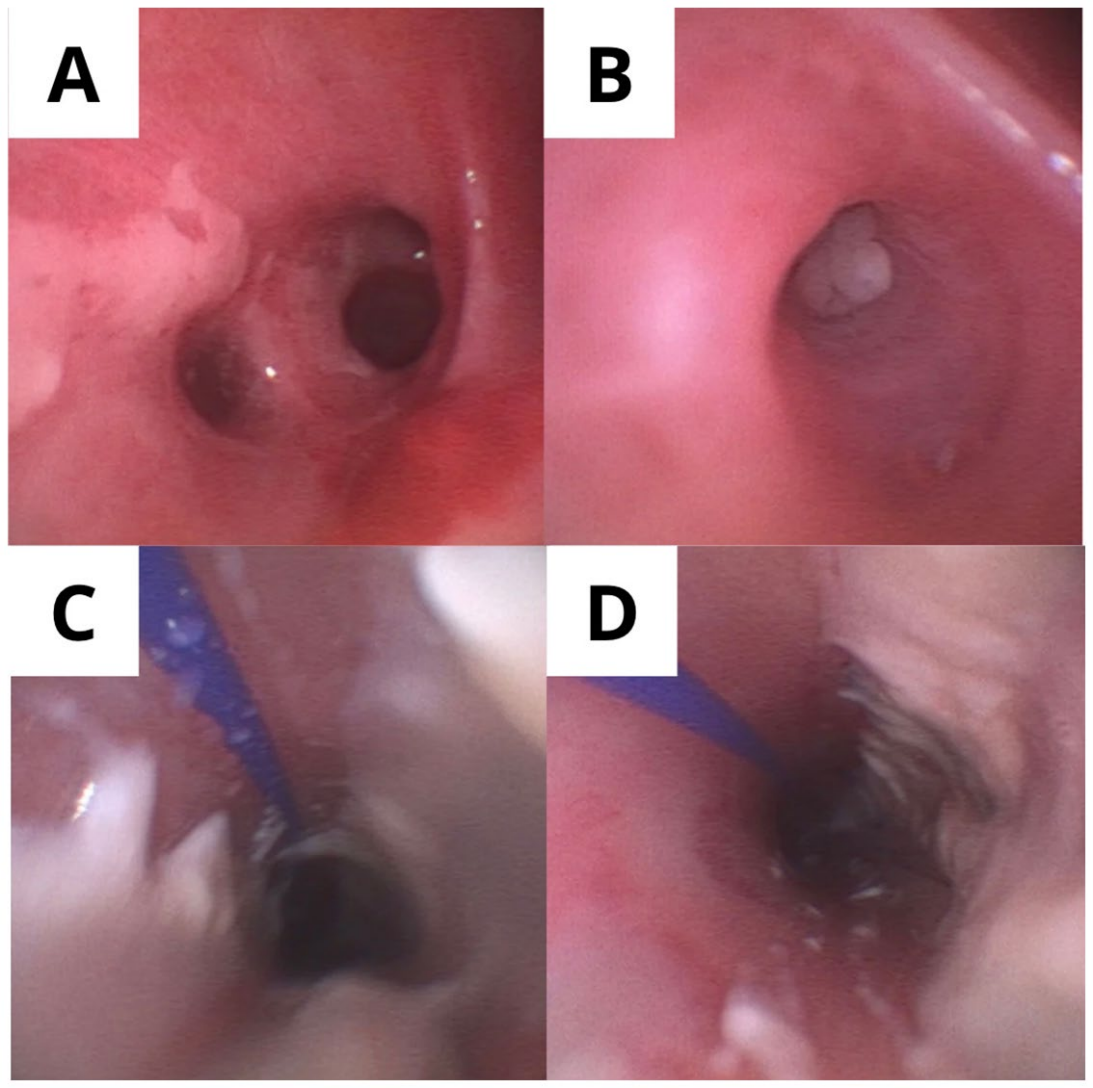
Chest CT imaging. **(A)** Day 2: Rounded bilateral consolidations with cylindrical bronchiectasis and air trapping, predominantly in the left lower lobe. **(B)** Day 18: Resolution of previous findings with normal parenchymal appearance. Serial chest computed tomography demonstrating radiologic evolution. Axial chest CT images in lung window (W: 1,500, L: −600) at the level of the lower thorax. **(A)** Hospital day 2 (post-intubation): Multifocal rounded consolidations with air bronchograms (white arrow) are visible bilaterally, with predominant involvement of the left lower lobe. Cylindrical bronchiectasis (arrowhead) is present in the basal segments, and mosaic attenuation with air trapping (darker regions) suggests small airway obstruction. These findings, combined with multiplex PCR detection of parainfluenza virus, rhinovirus, *Haemophilus influenzae*, *Staphylococcus aureus*, and *Moraxella catarrhalis* from bronchoalveolar lavage performed on the same day, confirmed severe polymicrobial pneumonia as a major precipitant of diabetic ketoacidosis. **(B)** Hospital day 18 (pre-discharge): Complete resolution of consolidations with restoration of normal parenchymal architecture and aeration. The radiologic recovery paralleled clinical improvement, with successful extubation on day 4 and ICU discharge on day 7. This favorable evolution demonstrates the reversibility of acute lung injury in the setting of ketosis-prone diabetes with severe infection when appropriate antimicrobial therapy and supportive care are provided. Images were acquired without intravenous contrast due to acute kidney injury at presentation (creatinine 1.4 mg/dL, subsequently normalized). CT protocol: Non-contrast helical CT; slice thickness 1.25 mm; tube voltage 120 kVp; automatic tube current modulation.

Chest X-ray was non-revealing. Chest CT on hospital day 2 revealed multifocal consolidations, cylindrical bronchiectasis, and air trapping predominantly in the left lower lobe (Figures 1A–D). She was admitted to the intensive care unit and initiated on high-flow oxygen and protocolized DKA management. DKA management was initiated with a 1-L bolus of 0.9% sodium chloride over the first hour, followed by intravenous fluids at 250–300 mL/h for ongoing volume repletion. After initial stabilization, fluids were transitioned to balanced crystalloids (Ringer’s lactate) to mitigate hyperchloremic metabolic acidosis and support more efficient correction of acid–base derangements, in line with contemporary evidence favoring balanced crystalloids over normal saline in DKA (15). Continuous intravenous insulin infusion was started at 0.1 U/kg/h without an initial bolus, with hourly point-of-care glucose monitoring. Once plasma glucose decreased below 250 mg/dL, dextrose-containing fluids were initiated to permit continued insulin administration until resolution of ketoacidosis. Serum potassium at presentation was 4.2 mmol/L; potassium supplementation (chloride and/or acetate salts) was titrated with 4–6 hourly electrolyte monitoring to maintain serum potassium between 4.0 and 5.0 mmol/L, following current inpatient diabetes care standards (16). Due to respiratory deterioration, endotracheal intubation and mechanical ventilation were required within 6 h.

**Figure 2:**
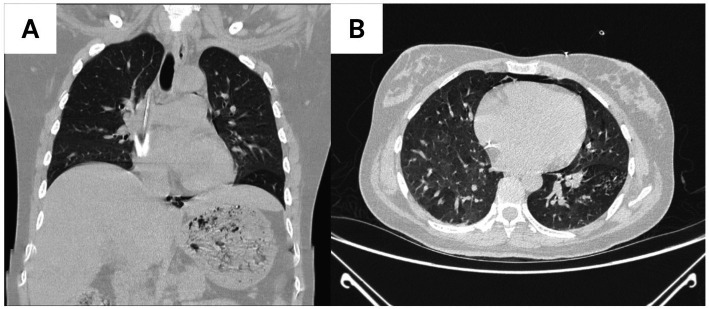
Bronchoscopic appearance during bronchoalveolar lavage (day 2). **(A)** Thick purulent secretions obstructing the segmental bronchus of the left lower lobe. **(B)** Erythematous bronchial mucosa with mucopurulent exudate and adherent whitish, curd-like plaques consistent with a pseudomembranous appearance. Bronchoscopic documentation of severe tracheobronchial inflammation and candidiasis. Fiberoptic bronchoscopic images obtained during bronchoalveolar lavage on ICU day 2. **(A)** Left lower lobe segmental bronchus showing thick, yellow-green purulent secretions (asterisk) causing partial airway obstruction. The volume and tenacity of secretions necessitated frequent suctioning and contributed to ventilatory difficulty. **(B)** After partial clearance of secretions, the underlying bronchial mucosa is markedly erythematous and hyperemic, with prominent vascular engorgement consistent with acute inflammation. Distinct white, curd-like plaques (white arrows) are firmly adherent to the mucosa, creating a pseudomembranous appearance characteristic of tracheobronchial candidiasis. These plaques could not be easily removed with gentle suctioning, distinguishing them from superficial secretions. Bronchoalveolar lavage fluid from this segment grew *Candida albicans* in culture, and serum *Candida* mannan antigen was positive. This integrated endoscopic and microbiologic evidence supported the diagnosis of invasive *Candida* tracheobronchitis rather than simple colonization, justifying the initiation of fluconazole therapy (400 mg IV daily for 14 days) in accordance with clinical criteria for tracheobronchial fungal infection in critically ill patients. Bronchoscopy details: Flexible fiberoptic bronchoscopy performed via 7.5 mm endotracheal tube using Olympus BF-1 T180 bronchoscope; BAL performed with 3 × 50 mL aliquots of sterile 0.9% saline in left lower lobe basilar segment; return volume 65 mL (43%); samples sent for bacterial/fungal culture, multiplex PCR (BioFire FilmArray RP2plus Pneumonia Panel), and cytology. BAL, bronchoalveolar lavage; ICU, intensive care unit; IV, intravenous.

Bronchoscopy with bronchoalveolar lavage (BAL) on ICU day 2 showed thick mucus, erythematous mucosa, and purulent secretions. Distinct white, curd-like plaques firmly adherent to the bronchial mucosa were observed, consistent with a pseudomembranous appearance (Figures 2A,B). Multiplex PCR (BioFire RP2plus) identified parainfluenza virus, rhinovirus/enterovirus, *Haemophilus influenzae* (10^6^ copies/mL), *Staphylococcus aureus* (>10^7^ copies/mL, high burden), and *Moraxella catarrhalis*. *Candida albicans* was cultured. Serum Candida mannan antigen was positive; serum β-D-glucan testing was not available at our institution. Fluconazole was initiated for 14 days based on the endoscopic pseudomembranous lesions and supportive serologic evidence, consistent with reported features of tracheobronchial candidiasis (17, 18).

Empirical antibiotics were adjusted to cefuroxime and clindamycin. The patient remained on mechanical ventilation (tidal volume 6 mL/kg, PEEP 8 cm H₂O, FiO₂ 0.6) for 96 h. Biochemical resolution was defined using standard criteria (bicarbonate ≥15 mEq/L, pH > 7.30, and anion gap ≤12 mEq/L, with glucose <250 mg/dL while receiving dextrose-containing fluids) (16). The anion gap decreased from 26 mEq/L at presentation to the normal range by approximately 36 h, with pH > 7.30 achieved by hour 28. Intravenous insulin was then transitioned to a subcutaneous basal–bolus regimen after confirmation of anion-gap closure and tolerance of oral intake, using appropriate overlap to prevent rebound ketosis (16). She was extubated and discharged from the ICU on day 7.

She completed a 14-day hospital stay and was discharged on insulin therapy. At one-month follow-up, HbA1c had decreased to 7.2%. Insulin was progressively tapered and discontinued by month five, with HbA1c of 5.6%. Three months after insulin discontinuation, repeat HbA1c was 5.4%, and a 75-g oral glucose tolerance test demonstrated normal glucose tolerance (fasting 92 mg/dL; 2-h 130 mg/dL), supporting metabolic remission and aligning with current recommendations for post-DKA phenotyping with glucose-based testing (19, 20).

Pulmonary function tests at 1 month showed a mild restrictive defect and reduced diffusing capacity for carbon monoxide (DLCO 70%). At 6 months, all parameters had normalized (DLCO 99%). Cardiopulmonary exercise testing revealed a ventilatory equivalent for carbon dioxide (VE/VCO₂) of 35 at 1 month, improving to 30 at 6 months. Key investigations and therapeutic milestones are summarized in Tables 1–3.

**Table 1:** Chronology of clinical evolution, diagnostic milestones, key investigations, and therapeutic interventions (including DKA management and follow-up glycemic assessment).

Time point	Clinical event	Key laboratory/imaging findings	Diagnostic procedures	Therapeutic interventions
Day 0 (Admission)	ED presentation: altered mental status, tachypnea, dehydration	pH 7.09, glucose 548 mg/dL, AG 26 mEq/L, HCO₃⁻ 6.1 mmol/L, HbA1c 9.0%, WBC 17,890/μL, CRP 25.3 mg/L, SpO₂ 89% RA	–	ICU admission; DKA protocol: 1-L 0.9% NaCl bolus, then 250–300 mL/h; IV insulin 0.1 U/kg/h; High-flow O₂
Hour 6	Progressive respiratory failure	PaO₂/FiO₂ < 200, RR 35/min, increasing work of breathing	–	Endotracheal intubation; MV (TV 6 mL/kg, PEEP 8, FiO₂ 0.6)
Hour 28	Metabolic improvement	pH > 7.30 achieved, HCO₃⁻ 14.2 mmol/L	–	Continue IV insulin + dextrose-containing fluids; Transition to balanced crystalloids (Ringer’s lactate)
Hour 36	Anion gap closure	AG ≤ 12 mEq/L, HCO₃⁻ 16 mmol/L, glucose 180 mg/dL	–	Plan transition to subcutaneous insulin
Day 2 (ICU day 2)	Bronchoscopy performed	Chest CT: multifocal consolidations, bronchiectasis, air trapping (left lower lobe)	BAL: Multiplex PCR: parainfluenza, rhinovirus, *H. influenzae*, *S. aureus* (high burden), *M. catarrhalis*; Culture: *C. albicans*; Mannan Ag (+)	Antibiotics: cefuroxime 750 mg IV q8h + clindamycin 600 mg IV q8h; Fluconazole 400 mg IV daily initiated
Day 3	Insulin transition	Oral intake tolerated, ketones cleared, glucose 140–180 mg/dL	Diabetes autoantibodies: GAD65 (−), IA-2 (−), ZnT8 (−); C-peptide 2.1 ng/mL	Subcutaneous basal-bolus insulin (glargine 10 U qHS + lispro 4 U pre-meals)
Day 4	Successful extubation	SpO₂ 96% on 2 L NC, RR 18/min, hemodynamically stable	–	Transfer to step-down unit; Continue antibiotics/fluconazole
Day 7	ICU discharge	Clinically stable, afebrile, glucose 100–150 mg/dL	–	Transfer to general medical ward
Day 14	Hospital discharge	Antibiotics/fluconazole completed (14 days each)	–	Discharge on insulin: glargine 8 U qHS + lispro 3 U pre-meals
Day 18	–	Repeat chest CT: complete resolution of consolidations	–	–
1 Month	Follow-up visit	HbA1c 7.2%; PFTs: DLCO 70%, mild restriction	Pulmonary function testing	Insulin dose reduced: glargine 6 U qHS + lispro 2 U pre-meals
3 Months	Follow-up visit	HbA1c 6.1%, fasting glucose 100–110 mg/dL	–	Further insulin taper: glargine 4 U qHS + lispro 1 U pre-meals
5 Months	Insulin discontinuation	HbA1c 5.6%, fasting glucose 90–100 mg/dL, PPG < 140 mg/dL on CGM	Continuous glucose monitoring	Insulin discontinued; Diet + exercise management
6 Months	Follow-up visit	PFTs normalized: DLCO 99%, TLC 97%; VE/VCO₂ 30	Pulmonary function + cardiopulmonary exercise testing	Continue lifestyle modifications
8 Months	Metabolic phenotyping	HbA1c 5.4%; OGTT: fasting 92 mg/dL, 2-h 130 mg/dL (normal tolerance)	75-g oral glucose tolerance test	Diagnosis: ketosis-prone diabetes with remission confirmed

ED, emergency department; ICU, intensive care unit; DKA, diabetic ketoacidosis; AG, anion gap; HCO₃⁻, bicarbonate; RA, room air; IV, intravenous; MV, mechanical ventilation; TV, tidal volume; PEEP, positive end-expiratory pressure; BAL, bronchoalveolar lavage; PCR, polymerase chain reaction; Ag, antigen; NC, nasal cannula; PFTs, pulmonary function tests; DLCO, diffusing capacity for carbon monoxide; PPG, postprandial glucose; CGM, continuous glucose monitoring; TLC, total lung capacity; VE/VCO₂, ventilatory equivalent for carbon dioxide; OGTT, oral glucose tolerance test.

DKA management was initiated according to contemporary inpatient diabetes care standards, including protocolized insulin infusion, potassium repletion, and transition to balanced crystalloids after initial stabilization to mitigate hyperchloremic acidosis and support more efficient metabolic recovery. Follow-up glycemic assessment included structured phenotyping with oral glucose tolerance testing to confirm metabolic remission and clarify long-term risk profile.

**Table 2:** Pulmonary function and cardiopulmonary exercise recovery timeline and interpretation.

Parameter	Reference range	1 month post-discharge	6 months post-discharge	Interpretation
Spirometry				
FVC (L)	3.2–4.5	2.8 (78% predicted)^ᵃ^	3.9 (95% predicted)	Complete recovery of lung volumes
FEV₁ (L)	2.8–3.9	2.4 (76% predicted)^ᵃ^	3.5 (94% predicted)	Resolution of restrictive pattern
FEV₁/FVC ratio	>0.70	0.86	0.90	No obstructive component at any time point
Lung volumes				
TLC (L)	4.5–6.0	4.2 (82% predicted)^ᵃ^	5.3 (97% predicted)	Normalization of total lung capacity
RV (L)	1.2–1.8	1.4 (89% predicted)	1.5 (94% predicted)	Normal residual volume throughout
Gas exchange				
DLCO (mL/min/mmHg)	20–30	14.8 (70% predicted)^ᵃ^	21.2 (99% predicted)	Marked improvement in diffusion capacity
DLCO/VA	4.0–6.0	3.2^ᵃ^	4.8	Resolution of alveolar-capillary impairment
Exercise testing				
Peak VO₂ (mL/kg/min)	>25	18.4 (65% predicted)^ᵃ^	26.8 (94% predicted)	Restoration of aerobic capacity
VE/VCO₂ slope	25–35	35^ᵃ^	30	Improved ventilatory efficiency
Peak HR (bpm)	190–195	168	188	Normal chronotropic response at 6 months
SpO₂ at peak (%)	>95	94^ᵃ^	98	No exercise-induced desaturation at 6 months

FVC, forced vital capacity; FEV₁, forced expiratory volume in 1 s; TLC, total lung capacity; RV, residual volume; DLCO, diffusing capacity for carbon monoxide; VA, alveolar volume; VO₂, oxygen consumption; VE/VCO₂, ventilatory equivalent for carbon dioxide; HR, heart rate; SpO₂, oxygen saturation.

ᵃ Indicates values below normal range.

Pulmonary recovery was evaluated at 1 and 6 months, showing resolution of restriction/diffusion impairment and improved ventilatory efficiency after severe pneumonia and critical illness. Reference ranges are based on prediction equations for a 28-year-old woman with height 165 cm and weight 67 kg (BMI 24.8 kg/m^2^).

At 3 months after insulin discontinuation, HbA1c was 5.4% and OGTT was normal (fasting 92 mg/dL; 2-h 130 mg/dL), supporting metabolic remission. This parallel resolution of respiratory and metabolic derangements underscores the reversibility of both organ system dysfunctions with appropriate acute management and structured follow-up.

**Table 3:** Essential investigations and diagnostic/treatment timeline.

Investigation category	Timing	Key findings	Clinical significance
Initial laboratory assessment	Day 0 (admission)	pH 7.09, glucose 548 mg/dL, AG 26 mEq/L, HbA1c 9.0%, WBC 17,890/μL, CRP 25.3 mg/L	Confirmed severe DKA with marked inflammatory response
Diabetes phenotyping	Day 3	GAD65 (−), IA-2 (−), ZnT8 (−); C-peptide 2.1 ng/mL	Excluded autoimmune type 1 diabetes; preserved endogenous insulin secretion
Respiratory imaging	Day 2	Chest CT: multifocal consolidations, cylindrical bronchiectasis, air trapping (left lower lobe)	Documented severe pneumonia as precipitant
Microbiologic diagnosis	Day 2 (BAL)	Multiplex PCR: parainfluenza, rhinovirus, *H. influenzae*, *S. aureus* (high), *M. catarrhalis*; Culture: *C. albicans*; Mannan Ag (+)	Confirmed polymicrobial bacterial/viral pneumonia + invasive candidiasis
Antimicrobial therapy	Days 2–14	Cefuroxime + clindamycin × 14 days; Fluconazole 400 mg IV daily × 14 days	Targeted therapy based on stewardship-informed interpretation
DKA resolution	Hour 36	AG ≤ 12 mEq/L, pH > 7.30, HCO₃⁻ 16 mmol/L	Met biochemical criteria for DKA resolution
Radiologic follow-up	Day 18	Chest CT: complete resolution of consolidations	Confirmed radiologic recovery
Pulmonary function assessment	1 month, 6 months	Month 1: DLCO 70%, mild restriction; Month 6: DLCO 99%, all parameters normalized	Documented complete pulmonary recovery
Long-term glycemic assessment	8 months	HbA1c 5.4%; OGTT: fasting 92 mg/dL, 2-h 130 mg/dL (normal)	Confirmed ketosis-prone diabetes with sustained remission

DKA, diabetic ketoacidosis; AG, anion gap; WBC, white blood cell count; CRP, C-reactive protein; GAD65, glutamic acid decarboxylase 65 antibody; IA-2, insulinoma-associated protein 2 antibody; ZnT8, zinc transporter 8 antibody; CT, computed tomography; BAL, bronchoalveolar lavage; PCR, polymerase chain reaction; Ag, antigen; IV, intravenous; HCO₃⁻, bicarbonate; DLCO, diffusing capacity for carbon monoxide; OGTT, oral glucose tolerance test.

DKA management aligned with ADA inpatient standards and contemporary evidence supporting balanced crystalloids in DKA. Antimicrobial therapy was guided by stewardship-based interpretation of multiplex PCR results, integrating pathogen burden, clinical syndrome, and radiologic findings.

## Discussion

This case illustrates how severe polymicrobial pneumonia and recent high-altitude exposure can precipitate diabetic ketoacidosis in a patient with previously unrecognized chronic dysglycemia. The coexistence of multiple respiratory pathogens, critical illness, and stress-related metabolic decompensation aligns with the concept that polymicrobial infections in critically ill hosts amplify inflammatory responses and increase the risk of severe systemic complications (21).

High-altitude exposure likely contributed to metabolic vulnerability through multiple mechanisms. Acute hypobaric hypoxia triggers sympathetic nervous system activation and counter-regulatory hormone surges, promoting insulin resistance and enhanced lipolysis with subsequent ketogenesis (3–5). At the cellular level, hypoxia-inducible factor signaling may directly impair pancreatic β-cell function and insulin secretion (7). Additionally, altitude-associated dehydration and altered immune responses may have lowered the threshold for both respiratory infection and ketoacidosis in this patient with underlying but previously unrecognized dysglycemia (6, 8, 11). This case thus exemplifies how environmental stressors can unmask latent metabolic disease through convergent pathophysiological mechanisms.

The broad pathogen spectrum detected by multiplex molecular testing, including respiratory viruses and typical bacterial respiratory pathogens, highlights both the diagnostic power and interpretive challenges of these platforms in lower respiratory tract infections. “Real-world evaluations of the BioFire FilmArray Pneumonia Panel show that it can substantially enhance pathogen detection compared with culture and inform antimicrobial optimization but also emphasize that colonization and upper-airway carriage are frequently detected and must be distinguished from true infection (22–24). Studies of panel performance in lower respiratory tract samples confirm good sensitivity and specificity, yet stress that results require integration with clinical, radiologic, and quantitative data to support stewardship-focused decisions (23).

The persistence and kinetics of respiratory virus detection further complicate interpretation in complex cases like this one. Data on prolonged viral PCR positivity show that nucleic acid from respiratory viruses can remain detectable beyond the period of peak symptoms, particularly in hospitalized patients, meaning that a positive PCR does not always indicate active, causative infection at the time of testing (25). This reinforces the need to prioritize high-burden bacterial targets and the overall clinical syndrome when deciding which organisms to treat in polymicrobial reports (22, 23, 25).

The identification of *Candida albicans* in bronchoalveolar lavage in an ICU patient with DKA and mechanical ventilation raises the classic dilemma of colonization versus invasive disease. Reviews on invasive candidiasis in the ICU emphasize that Candida isolated from respiratory secretions usually reflects colonization and that antifungal therapy should be reserved for situations with supportive clinical, endoscopic, or serologic evidence of invasive tracheobronchial or parenchymal disease (17). Case series of Candida tracheobronchitis describe pseudomembranous, plaque-like lesions adherent to the bronchial mucosa, often accompanied by systemic or local indicators of fungal infection and favorable responses to antifungal therapy, providing a rationale for targeted treatment in selected patients such as the one presented (18).

Beyond the acute episode, accurate classification and long-term risk assessment are critical in patients whose first presentation of diabetes is DKA. Contemporary standards of care for diagnosis and classification of diabetes underscore that HbA1c alone may miss some forms of dysglycemia and that classification should integrate clinical phenotype, autoantibody status, and, when appropriate, additional testing (19). A systematic review and meta-analysis in stroke populations demonstrates that oral glucose tolerance testing can identify diabetes and impaired glucose regulation that are not detected by HbA1c, supporting the use of OGTT when precise characterization of glucose tolerance is needed, as in suspected ketosis-prone diabetes after metabolic remission (20).

Although the admission HbA1c of 9.0% indicates sustained hyperglycemia over the preceding 8–12 weeks, thereby excluding isolated stress-induced hyperglycemia in an otherwise metabolically normal individual, several key findings support ketosis-prone diabetes rather than autoimmune type 1 diabetes or established symptomatic type 2 diabetes. The absence of diabetes autoantibodies, preserved C-peptide secretion, and subsequent insulin independence are characteristic of ketosis-prone diabetes with remission. We interpret this presentation as diabetic ketoacidosis precipitated by acute physiological stressors—specifically high-altitude exposure and severe infection—occurring in a patient with previously unrecognized chronic dysglycemia. This clinical phenotype aligns with current descriptions of A^−^β^+^ ketosis-prone diabetes, a distinct entity in which patients present with DKA but lack islet autoimmunity and retain sufficient pancreatic β-cell reserve to achieve insulin independence following resolution of the acute metabolic crisis (2, 10, 26, 27).

The distinction between stress-induced hyperglycemia and pre-existing chronic dysglycemia unmasked by acute stress is clinically and prognostically important. True stress hyperglycemia occurs in previously normoglycemic individuals during critical illness and typically resolves once the acute stressor is removed, whereas sustained HbA1c elevation reflects months of antecedent dysglycemia and indicates underlying diabetes or prediabetes (28, 29). In this case, the admission HbA1c of 9.0%, combined with subsequent demonstration of glucose intolerance requiring insulin therapy before eventual remission, supports the interpretation that altitude exposure and infection precipitated ketoacidosis in the setting of previously unrecognized chronic hyperglycemia rather than inducing transient hyperglycemia in a metabolically normal host.

Finally, the multiplicity of pathogens observed in this case fits with broader evidence that polymicrobial infections in critically ill hosts are associated with more severe clinical courses and complex therapeutic decisions (21). The patient’s full recovery of pulmonary function and normalization of glucose tolerance after withdrawal of insulin therapy illustrates that, with timely diagnosis, judicious use of molecular diagnostics, and structured metabolic follow-up, both respiratory and metabolic derangements can be reversible even in severe presentations.

This case report has several limitations. As a single-patient observation, generalizability is limited. Pathogen quantification from multiplex PCR was semiquantitative, and absolute burden thresholds for clinical significance remain undefined, and comparative studies show variability in panel performance across different clinical settings (30). Serum β-D-glucan testing was unavailable (30) Serum β-D-glucan testing was unavailable, potentially limiting diagnostic certainty for invasive candidiasis. Although 8-month follow-up demonstrated sustained remission, longer observation is needed to confirm durability and exclude late relapse. Finally, genetic testing for monogenic diabetes was not pursued given low clinical suspicion, but cannot be definitively excluded.

## Conclusion

This case of diabetic ketoacidosis precipitated by high-altitude exposure and severe polymicrobial pneumonia in a previously undiagnosed young woman highlights the interplay between environmental stressors, complex respiratory infection, and ketosis-prone diabetes. A careful, stewardship-based interpretation of multiplex pneumonia panel results was essential to distinguish likely pathogenic organisms from colonizers, thereby guiding antimicrobial and antifungal therapy while minimizing unnecessary treatment.

Structured post-discharge evaluation, including classification according to current diabetes standards and the use of oral glucose tolerance testing, confirmed metabolic remission and clarified the long-term phenotype and risk profile. This integrated respiratory, infectious, and metabolic approach can inform the management of similar patients who present with combined respiratory failure and DKA in the context of recent high-altitude exposure and polymicrobial airway infection.

## Data Availability

The raw data supporting the conclusions of this article will be made available by the authors, without undue reservation.
